# RETRACTION: Enhanced Recovery After Surgery Care to Reduce Surgical Site Wound Infection and Postoperative Complications for Patients Undergoing Liver Surgery

**DOI:** 10.1111/iwj.70151

**Published:** 2024-12-05

## Abstract

**RETRACTION:** WangY.‐L. , ZhangF.‐B. , ZhengL.‐E. , YangW.‐W. , and KeL.‐L. , “Enhanced Recovery After Surgery Care to Reduce Surgical Site Wound Infection and Postoperative Complications for Patients Undergoing Liver Surgery,” International Wound Journal 21, no. 4 (2024): e14832, 10.1111/iwj.14227.39637867

The above article, published online on 22 May 2024, in Wiley Online Library (http://onlinelibrary.wiley.com/), has been retracted by agreement between the journal Editor in Chief, Professor Keith Harding; and John Wiley & Sons Ltd. Following an investigation by the publisher, all parties have concluded that this article was accepted solely on the basis of a compromised peer review process. The editors have therefore decided to retract the article. The authors did not respond to our notice regarding the retraction.

**RETRACTION:** Y.‐L. Wang , F.‐B. Zhang , L.‐E. Zheng , W.‐W. Yang , and L.‐L. Ke , “Enhanced Recovery After Surgery Care to Reduce Surgical Site Wound Infection and Postoperative Complications for Patients Undergoing Liver Surgery,” International Wound Journal 21, no. 4 (2024): e14832, 10.1111/iwj.14227.39637867

The above article, published online on 22 May 2024, in Wiley Online Library (http://onlinelibrary.wiley.com/), has been retracted by agreement between the journal Editor in Chief, Professor Keith Harding; and John Wiley & Sons Ltd. Following an investigation by the publisher, all parties have concluded that this article was accepted solely on the basis of a compromised peer review process. The editors have therefore decided to retract the article. The authors did not respond to our notice regarding the retraction.

