# Correction to Retraction: Enhanced Recovery After Surgery Care to Reduce Surgical Site Wound Infection and Postoperative Complications for Patients Undergoing Liver Surgery

**DOI:** 10.1111/iwj.70174

**Published:** 2025-02-09

Correction: https://doi.org/10.1111/iwj.70151.

The following retraction, published online on 05 December 2024 [https://doi.org/10.1111/iwj.70151], has been amended, and the online version has been revised to reflect the correction. The publication date and page numbers for the retracted article were incorrectly listed in the original retraction statement and have been revised in the new version.

